# Current cannabis use and pain management among US cancer patients

**DOI:** 10.1007/s00520-024-08321-9

**Published:** 2024-01-18

**Authors:** Jessica L. Krok-Schoen, Jesse J. Plascak, Alison M. Newton, Scott A. Strassels, Anita Adib, Neema C. Adley, John L. Hays, Theodore L. Wagener, Erin E. Stevens, Theodore M. Brasky

**Affiliations:** 1 School of Health and Rehabilitation Sciences, The Ohio State University College of Medicine, Columbus, OH USA; 2 Division of Cancer Prevention and Control, The Ohio State University College of Medicine, Columbus, OH USA; 3 Division of Medical Oncology, The Ohio State University College of Medicine-James Comprehensive Cancer Center, 420 W. 12th Ave., Suite 514B TRF, Columbus, OH 43210 USA; 4 Atrium Health, Charlotte, NC USA; 5 Department of Neuroscience, The Ohio State University College of Arts and Sciences, Columbus, OH USA; 6 Department of Internal Medicine, The Ohio State University College of Medicine, Columbus, OH USA

**Keywords:** Cancer, Cannabis, Epidemiology, Marijuana, Prevalence

## Abstract

**Abstract:**

**Background:**

National studies reporting the prevalence of cannabis use have focused on individuals with a history of cancer without distinction by their treatment status, which can impact symptom burden. While pain is a primary motivation to use cannabis in cancer, the magnitude of its association with cannabis use remains understudied.

**Methods:**

We examined cannabis use and pain management among 5523 respondents of the Behavioral Risk Factor Surveillance System with a cancer history. Survey-weighted prevalence proportions of respondents’ cannabis use are reported, stratified on cancer treatment status. Regression models estimated odds ratios (ORs) and 95% confidence intervals (CIs) of cancer-related pain and cannabis use.

**Results:**

Cannabis use was slightly more prevalent in those undergoing active treatment relative to those who were not undergoing active treatment (9.3% vs. 6.2%; *P*=0.05). Those under active treatment were more likely to use cannabis medicinally (71.6% vs. 50.0%; *P*=0.03). Relative to those without cancer-related pain, persons with pain under medical control (OR 2.1, 95% CI, 1.4–3.2) or uncontrolled pain were twice as likely to use cannabis (OR 2.0, 95% CI, 1.1–3.5).

**Conclusions:**

Use of cannabis among cancer patients may be related to their treatment and is positively associated with cancer-related pain. Future research should investigate the associations of cannabis use, symptom burden, and treatment regimens across the treatment spectrum to facilitate interventions.

## Introduction

Approximately 50 million Americans (18%) aged ≥12 years have used cannabis products in the past year [1]. The 2020 National Survey on Drug Use and Health reported past-year cannabis use increased from 7% of respondents in 2002 to 16% in 2020 [1].

Individuals with cancer use cannabis products to manage pain and other symptoms of cancer and its treatment [2]. Patients report that cannabis provides a relatively high level of symptom relief [2], yet nationally representative data among oncologists indicate significant uncertainty on cannabis’ effectiveness to treat pain alone or as an adjunct to palliative therapies [3]. The prevalence of cannabis use by treatment status is not well understood. In nationally representative estimates, about 7–10% of persons with a history of cancer report using cannabis [4–9]. Estimates of use in individuals under active cancer care are generally derived from single-institution studies and range from 8 to 25% [2, 10–16]. There is, therefore, an implication of higher cannabis prevalence among cancer patients receiving treatment, yet no study using representative sampling has made a direct comparison, leaving estimate differences poorly explained. While pain is among the top reasons for cannabis use [2], there remains little understanding of the relation between pain and use of cannabis products among people with cancer or whether such associations differ by treatment status.

The purpose of this study was to investigate differences in cannabis use and pain management behaviors by treatment status among cancer populations among respondents of a national study.

## Methods

The Behavioral Risk Factor Surveillance System (BRFSS) is administered annually as a telephone-based survey and designed to represent residents’ health-related behaviors and health conditions [17]. Different from prior reports using BRFSS data [4, 5, 7], we restricted our analysis to adults with cancer residing in the nine states (Delaware, Hawaii, Indiana, Mississippi, Montana, Rhode Island, South Carolina, Utah, and West Viriginia) that employed optional cannabis use and cancer survivorship modules in 2020 or 2021 and made their data available to the CDC. The cancer survivorship module identifies respondents’ primary cancer site/type, treatment status, age at diagnosis, and cancer pain. At the time of data collection, cannabis was fully legal in Rhode Island, Delaware, and Montana (2021); medically legal in Hawaii, Utah, Montana (2020), and West Virgina; and illegal in Indiana, South Carolina, and Mississippi. Response rates ranged between 38.5% and 67.2%.

Recent cannabis use was classified as “yes” if respondents indicated cannabis use in the past 30 days. Participants’ reasons for cannabis use were classified “medical,” “non-medical,” and “both medical and non-medical” from the questionnaire item, “When you used marijuana or cannabis during the past 30 days, was it usually:,” limited to those classified as “yes” for recent cannabis use. Participants were additionally asked whether they were receiving treatment for their cancer. Those who indicated in the affirmative were considered actively treated. Those who indicated that they were not receiving treatment for any reason (completed treatment, refused treatment, etc.) were considered to be inactively treated. Participants’ cancer-related pain was classified as none, controlled without medication, controlled with medication, and uncontrolled pain.

We report survey-weighted frequencies and proportions of participants’ clinical and sociodemographic characteristics overall and stratified on cancer treatment status. Differences in cannabis prevalence by cancer and treatment status were examined using chi-square tests. Zero inflated negative binomial (ZINB) regression models were used to estimate odds ratios (ORs) and 95% confidence intervals (95% CIs) of any cannabis use among users by cancer-related pain (none, controlled without medication, controlled with medication, uncontrolled pain), adjusted for participants’ sociodemographic, health behavior, clinical characteristics, and state-level cannabis legal status (see footnote to Table 3). We additionally report prevalence rate ratios and 95% CI of cannabis use frequency (i.e., days using in the past 30 days) by cancer-related pain categories (above) and from ZINB models. Models were fitted for all individuals and by treatment status. SAS version 9.4 (Cary, NC) was used to conduct all analyses. All statistical tests are two-sided, with a *P*<0.05 considered statistically significant.

## Results

Among 5523 BRFSS participants, representing 1,277,712 individuals with a cancer history residing in states that administered both the cannabis use and cancer survivorship questionnaires, 4557 (83.0%) were not receiving treatment and 966 (17.0%) were actively receiving treatment (Table 1). Most participants (52.9%) had reproductive cancers and reported no cancer-related pain (82.6%). The majority of sociodemographic characteristics, including participants’ age, sex, race/ethnicity, education, income, and geographic residence, were distributed equitably by strata of cancer treatment. However, actively treated patients were more likely to be out of work or unable to work compared to those not receiving treatment.

**Table 1:** Select sociodemographic characteristics stratified on cancer treatment status, US Behavioral Risk Factor Surveillance System 2020–2021 (*n*=5523)

	Overall, *n*=5523; *n* (%)	Treatment Status	
		Inactive, *n*=4557; *n* (%)	Active, *n*=996; *n* (%)
Age, years			
18–39	216 (7.4)	192 (8.2)	24 (3.5)
40–54	645 (16.3)	529 (16.0)	116 (17.7)
55–64	1060 (21.8)	857 (21.1)	203 (25.1)
≥65 years	3602 (54.6)	2979 (54.8)	623 (53.7)
Sex			
Male	2181 (40.6)	1759 (40.1)	422 (43.0)
Female	3342 (59.4)	2798 (59.9)	544 (57.0)
Marital status			
Married/unmarried couple	3091 (59.5)	2552 (59.0)	539 (61.7)
Separated/divorced/widowed	2028 (32.6)	1668 (32.5)	360 (32.9)
Never married	381 (7.9)	321 (8.4)	60 (5.4)
Race-ethnicity			
Non-Hispanic White	4445 (81.7)	3690 (82.2)	755 (79.2)
Non-Hispanic Black/African-American	371 (9.4)	291 (9.1)	80 (11.3)
Non-Hispanic Other	305 (4.1)	248 (4.0)	57 (4.4)
Non-Hispanic Multiracial	151 (1.9)	123 (1.9)	28 (2.3)
Hispanic/Latinx	141 (2.8)	113 (2.8)	28 (2.8)
Employment status			
Out of work/unable to work	727 (17.8)	552 (16.4)	175 (24.8)
Student/homemaker/retired	3299 (53.4)	2741 (54.0)	558 (50.4)
Employed	1477 (28.8)	1248 (29.6)	229 (24.8)
Education			
< High school	352 (12.7)	281 (12.2)	71 (15.0)
High school diploma or equivalent	1415 (29.0)	1198 (30.2)	217 (23.4)
Some college or technical school	1636 (32.6)	1344 (32.3)	292 (34.0)
≥ College or technical school	2107 (25.7)	1723 (25.3)	384 (27.6)
Annual income			
< $24,999	1002 (27.3)	835 (27.0)	167 (28.9)
$25,000–$49,999	1273 (29.2)	1042 (29.8)	231 (26.5)
$50,000–$74,999	811 (18.3)	650 (17.5)	161 (21.9)
≥ $75,000	1028 (25.2)	865 (25.7)	163 (22.6)
Geographic region of residence			
Northeast	964 (15.4)	178 (16.1)	786 (15.2)
South	539 (14.6)	89 (14.1)	450 (14.8)
Midwest	634 (32.2)	101 (30.1)	533 (32.6)
West	2495 (37.8)	439 (39.7)	2056 (37.5)
Body mass index, kg/m^2^			
<25	1579 (29.7)	1293 (29.1)	286 (32.9)
25–30	1860 (34.7)	1536 (34.9)	324 (33.3)
≥30	1776 (35.6)	1460 (36.0)	316 (33.8)
Tobacco smoking history			
Current	607 (15.8)	504 (16.2)	103 (14.1)
Former	1898 (35.0)	1558 (34.8)	340 (35.9)
Never	2984 (49.2)	2466 (49.1)	518 (50.0)
Cancer site/type group			
Female reproductive	1658 (36.4)	1412 (37.2)	246 (32.2)
Male reproductive	821 (16.5)	694 (16.9)	127 (14.6)
Gastrointestinal	444 (9.4)	374 (9.7)	70 (7.9)
Head and neck	63 (1.2)	47 (1.2)	16 (1.3)
Hematologic	271 (6.7)	191 (6.1)	80 (9.7)
Thoracic	352 (8.3)	293 (8.6)	59 (6.9)
Melanoma of the skin	200 (4)	164 (4.1)	36 (3.5)
Urinary	263 (5.6)	215 (5.3)	48 (7.1)
Other cancer	492 (11.9)	382 (10.9)	110 (16.8)
Cancer-related pain			
None	3521 (82.6)	3011 (86.1)	510 (65.6)
Controlled without medication	209 (5.9)	163 (5.7)	46 (7.0)
Controlled with medication	280 (8.1)	135 (4.9)	145 (23.3)
Uncontrolled pain	136 (3.4)	107 (3.3)	29 (4.1)
Medical marijuana legal status			
Legal	1173 (33.4)	983 (33.8)	190 (31.3)
Illegal	4350 (66.6)	3574 (66.2)	776 (68.7)

The prevalence of cannabis use overall was 6.8% (Table 2). However, this estimate differed by treatment status, with a lower prevalence (6.2%) among those not under active treatment, and a higher prevalence (9.3%) among actively treated patients. The difference was of borderline statistical significance (*P*=0.05). We additionally found that a greater proportion (71.6%) of actively treated patients reported using cannabis for medical reasons relative to those not actively receiving treatment (50.0%), and far fewer under active treatment used cannabis for non-medical reasons (7.1% vs. 20.8%; *P*=0.03). We did not observe a difference in the frequency of cannabis use by treatment status (*P*=0.13).

**Table 2:** Cannabis-related factors stratified on cancer recency and treatment status, US Behavioral Risk Factor Surveillance System 2020–2021 (*n*=5523)

	Overall *n*=5523; *n* (%)	Treatment status		*P* value^a^
		Inactive patients *n*=4557; *n* (%)	Active patients *n*=996; *n* (%)	
Recent cannabis product use				0.05
No	5158 (93.2)	4289 (93.8)	869 (90.7)	
Yes	337 (6.8)	249 (6.2)	88 (9.3)	
Past month cannabis use, days				0.13
None	5158 (93.2)	4289 (93.8)	869 (90.7)	
1–20	198 (4.0)	140 (3.7)	58 (5.9)	
21–30	139 (2.7)	109 (2.6)	30 (3.4)	
Cannabis use reason				0.03
Medical	198 (55.1)	140 (50.0)	58 (71.6)	
Non-medical	54 (17.6)	42 (20.8)	12 (7.1)	
Both	84 (27.4)	66 (29.2)	18 (21.2)	

^a^Derived from a chi-square test

The use of cannabis was positively associated with increased cancer-related pain (Table 3). Relative to those without cancer-related pain, those experiencing pain controlled with medication (adjusted OR 2.1, 95% CI 1.4–3.2) or uncontrolled pain (adjusted OR 2.0, 95% CI 1.1–3.5) had twice the odds of cannabis use. Although the magnitude of associations was higher among individuals actively receiving treatment, CIs were wide and included the null value. There was no association between cancer-related pain levels and the rate of cannabis use in the past 30 days (not shown).

**Table 3:** Associations of cancer-related pain with recent cannabis product use, US Behavioral Risk Factor Surveillance System 2020–2021 (*n*=5523)

	Overall		Treatment status	
	OR (95% CI)^a^, *n*=337	Adjusted OR (95% CI)^b^, *n*=337	Inactive OR (95% CI)^b^, *n*=249	Active OR (95% CI)^b^, *n*=88
Cancer-related pain				
None	1.0 referent	1.0 referent	1.0 referent	1.0 referent
Controlled without medication	1.9 (1.2–3.0)	1.4 (0.8–2.3)	0.8 (0.4–1.9)	5.4 (0.9–33.6)
Controlled with medication	2.6 (1.8–3.7)	2.1 (1.4–3.2)	1.8 (0.9–3.5)	1.6 (0.8–3.5)
Uncontrolled pain	3.5 (2.2–5.7)	2.0 (1.1–3.5)	1.8 (0.9–3.8)	2.7 (0.5–15.8)

^a^Unadjusted

^b^Adjusted for sex, age, marital status, race-ethnicity, employment status, education, body mass index, smoking status, cancer site, and state medical marijuana legal status

## Discussion

In this cross-sectional study representative of individuals residing in 9 US states, the prevalence of current cannabis use was nominally higher among individuals receiving cancer treatment compared to those who were not. Actively treated patients were additionally more likely to use cannabis medicinally. Lastly, cancer-related pain and current cannabis use were positively correlated.

Nearly all prior reports from national datasets, including the BRFSS [4–7], have been restricted to individuals with a history of cancer without distinction for treatment status. Among them, authors reported 7.4–10% cannabis use [4–9]. Similar to our findings, Cousins et al. [9] reported 10% cannabis prevalence among recent cancer patients (i.e., ≤12m since diagnosis) in the National Survey on Drug Use and Health. Although the likelihood of active treatment in that study is higher relative to reports including individuals with a cancer diagnosis decades prior, the authors did not explicitly examine cannabis use by treatment status. Treatment status may be an important factor given the high prevalence of symptom burden in patients across the cancer continuum [18] and the possibility that such patients may manage such symptoms with cannabis [2].

Pain was associated with higher likelihood of cannabis use. Individuals with cancer-related pain that was medically controlled or that was uncontrolled were 100–110% more likely to use cannabis than those without pain. Similarly, data from the Population Assessment of Tobacco and Health study reported that higher pain severity was associated with higher likelihood of cannabis use among individuals with a cancer history [8]. Our findings add to the literature on unresolved symptom burden, due in part to inadequate pain management, among cancer patients [18]. Cannabis may be a strategy to alleviate this burden, although clinical trial data on cannabis-based medicine for cancer pain are limited [2, 19–22]. Further research should investigate the associations of cannabis use, symptom burden, and treatment regimens across the treatment spectrum to identify where clinicians can intervene to improve their patient’s health-related quality of life.

Study limitations include reduced generalizability to states that included both the cancer survivorship and cannabis modules in their BRFSS data collection. We were further limited by a lack of detailed information about participants’ cancer history including cancer stage at diagnosis, detailed treatment data, and progression. The BRFSS is further limited by minimal measurement of cannabis product details (modes of use, methods of administration, etc.) and pain symptoms, symptom relief, and potential selection and response biases inherent to cross-sectional observational study designs. Lastly, this study included states with varying cannabis laws; however, sample sizes were too small to examine cannabis prevalence between them, especially when considering participants’ treatment status. Nevertheless, the effect of state laws on individuals’ cannabis use is not well understood. Brasky et al. [2] reported that only 27% of cancer patients using cannabis had a state-approved medicinal cannabis prescription in Ohio, a medical use state at the time. Additionally, a large percentage of those with a prescription combusted cannabis, which was, at the time, illegal in Ohio.

## Conclusion

Cannabis use was slightly higher among patients with cancer under active treatment compared to those who were not receiving treatment. Likewise, individuals under active cancer care were more likely to use cannabis medicinally. This study observed that pain was associated with higher likelihood of current cannabis use. Clinicians should facilitate discussions with their patients about cannabis-related behaviors and symptom management to support ongoing therapeutic relationships.

## Data Availability

The data used for this analysis are publicly available from the US Centers for Disease Control and Prevention.
